# Seafarers’ attitudes and chances to improve the nutrition on merchant ships from the crews’ and cooks’ perspective

**DOI:** 10.1186/s12995-024-00412-x

**Published:** 2024-05-02

**Authors:** Felix Alexander Neumann, Lukas Belz, Dorothee Dengler, Volker Harth, Chiara Reck, Marcus Oldenburg, Birgit-Christiane Zyriax

**Affiliations:** 1 Preventive Medicine and Nutrition, Midwifery Science - Health Services Research and Prevention, Institute for Health Services Research in Dermatology and Nursing (IVDP), University Medical Center Hamburg-Eppendorf (UKE), Hamburg, Germany; 2 Maritime Medicine, Institute for Occupational and Maritime Medicine Hamburg (ZfAM), University Medical Center Hamburg-Eppendorf (UKE), Hamburg, Germany

**Keywords:** Seafarer, Cook, Nutrition, Eating habits, Filipino, Burmese, European, Digital platform, Intervention measures

## Abstract

**Background:**

Seafarers’ diets are often high in fat, sugar and calories, thus contributing to an increased risk of obesity, metabolic syndrome and cardiovascular disease. The multitude of obstacles to healthy eating in the on-board environment on merchant ships makes it essential to find new approaches for health promotion. This study explored seafarers’ attitudes, the status quo of support measures and chances to improve nutrition on merchant ships from the perspective of crews and cooks.

**Methods:**

In the course of the EU-funded project “e-healthy ship”, European and Southeast Asian seafarers (*N* = 810) and ship cooks (*N* = 62) were examined by using two questionnaires on 68 ships of two German shipping companies.

**Results:**

Almost all seafarers (98.8%) considered a healthy diet important for their well-being and the majority of seafarers reported being open-minded about changing their eating habits (88.4%). However, European seafarers were less likely to respond that they are willing to eat less meat [OR 0.11; 95%CI (0.07–0.17); *p* < .001], more vegetables [OR 0.10; 95%CI (0.02–0.49); *p* = .005] and more fruits [OR 0.11; 95%CI (0.02–0.61); *p* = .011] than their Southeast Asian colleagues. On the one hand, 82.3% of the ship cooks reported having taken part in at least one cooking course organized by their employer (1: 33.9%, 2: 25.8%, 3: 14.5%, 4 or more: 8.1%), on the other hand, slightly above half stated that the last of these courses had taken place more than 2 years ago. Furthermore, the ship cooks showed a positive attitude towards the use of a tablet-based digital platform that supports the ship cooks in daily and complex tasks (> 85% agreement).

**Conclusions:**

To improve nutrition on board merchant ships, various parameters need to be adjusted, such as ensuring a demand-oriented food supply on board or supporting seafarers’ healthy food choices through target group-specific nutrition education. Ship cooks would be able to play a crucial role if they receive support. The development of a tablet-based digital platform that supports the ship cooks in their daily tasks, offers training and empowers them to implement health-promoting measures themselves seems to be an accepted and promising approach.

**Supplementary Information:**

The online version contains supplementary material available at 10.1186/s12995-024-00412-x.

## Introduction

An increased risk of overweight (body mass index [BMI] > 25 kg/m^2^), metabolic syndrome and cardiovascular disease has repeatedly been reported for seafarers on merchant ships [1–5]. In particular, seafarers’ diets, which tend to be high in fat, sugar, and calories [6], not only promote disease but also differ from the overall healthier diets of seafarers at home [7]. Therefore, the food on board undoubtedly plays an important role in the nutrition and health of seafarers. However, poor choice in food ordering, a mismatch between food ordering and deliveries by caterers, the limited accessibility of supplying ports and storage capacity on board, the varying shelf life of food and the difficulties of catering for mostly multinational crews are some of the factors that often give seafarers little opportunity to individually influence their diet over months of their stay on board [6, 8]. Seafarers’ decisions on individual consumption may be influenced by various factors, such as working conditions (e.g. time pressure, irregular working hours), climate (hot and dry weather), the changed living environment (e.g. ship movements, loneliness and isolation), but also fatigue and psychosocial factors which can ultimately lead to eating disorders (e.g. emotional eating, disinhibited eating behaviour) [9]. Although the importance of behavioural preventive measures to improve nutrition on board seems obvious [10], little is known about seafarers’ personal attitudes, in particular, the extent to which they are willing to change their eating habits (e.g. fruit, vegetable or meat consumption) on board still needs to be clarified. In addition, in the highly hierarchical system on board, the question arises to what extent healthy eating on the part of the superiors can have an impact on the crews.

The main task of the ship cooks is to provide healthy and tasty food. However, as they are usually part of a multinational crew and often have a different cultural background to some of the seafarers on board, it is difficult or impossible to take the preferences of the different nationalities into account and ensure that the food prepared meets the expectations of all seafarers. Additionally, the seafarers can use sweets and other sugary products which are available in special shops on board [8]. To date, no research has been conducted to find out, whether seafarers would also turn to healthy alternatives (such as nuts or pistachios) if they were offered.

While previous studies primarily examined the food supply of ships, as well as seafarers’ food and nutrient intake [6, 11], studies on the optimisation of the on-board food supply and the ship cooks in particular are still scarce. Only one study, which included 35 ship cooks from two Danish shipping companies, investigated whether the nutrition of seafarers on board could be improved by using a training intervention for the cooks [12]. Especially the less trained cooks benefited from the training in the long run, as they served more vegetables and fruit and implemented the tips on fat and sugar reduction from the course in a one-year follow-up. Good training of ship cooks therefore seems to be a promising way to improve the quality of food preparation. To improve the overall food supply, additional support is needed for food ordering, as this appeared to be overburdening for the ship cooks, who are mostly not trained for this.

Digital food environments are electronic interfaces through which people interact with a broader food system, enabling electronic ordering of food and beverages [13]. They have already been successful in improving users’ dietary patterns in various settings and were recently endorsed in a systematic review as a potential approach for the maritime setting [10]. The development of a digital platform delivered on a tablet that supports the ship cooks in daily and complex tasks, such as menu planning or food ordering, can be another innovative approach. Furthermore, by making the healthy choice the easy choice, suggested applying the principles of nudging, which can also be introduced to ship cooks on such a digital platform [14]. However, so far there is a lack of knowledge about whether the ship cooks would be open-minded to use digital tools for their work or to apply such intervention measures in practice.

Therefore, our study aimed to learn more about the food situation on board and how it can be improved, in particular by (a) the seafarers’ attitude towards healthy foods, (b) environmental factors on board that can impact seafarers’ food choice positively, (c) availability of possible support measures to improve meal quality for the cook, (d) the ship cooks’ attitudes towards interventional approaches such as the use of a tablet providing information and (e) nudging procedures applied by the cooks.

## Materials and methods

### Design and setting

The data collection was part of the EU-funded project “e-healthy ship”, which aimed to improve seafarers’ health and digitalise health management on board merchant ships. This study presents cross-sectional data from two questionnaires deployed on 68 ships with multinational crews from two German shipping companies in February 2019. The crew questionnaire addressed all seafarers on board except for the ship cooks and asked about attitudes, intentions and influencing factors towards their diet on board. The cook questionnaire was completed exclusively by cooks and asked about the availability of support measures for daily work as well as the willingness to perceive or implement support measures and intervention approaches to improve nutrition on board. Participation on this study was voluntary. Ethical approval was obtained from the Ethics Committee of the Hamburg Medical Association and the research was carried out in accordance with the Declaration of Helsinki.

### Processes and variables

The questionnaires were sent by email to the second officer of each ship, who printed them out and distributed them to the crew and cook. To ensure anonymity, the completed questionnaires were collected in sealed envelopes and returned to the research team by mail at the next port.

Demographic data asked for nationality, age, professional rank, type of ship, working area on the ship, self-reported body weight and height, as well as the job experience as a seafarer. In addition, the crew questionnaire asked seafarers to self-assess to several exploratory questions, such as, whether eating healthy foods is important for their well-being and whether they would be willing to change their eating habits on board. The seafarers were also asked whether superiors can serve as role models for healthy eating, whether satisfaction with the meals depends on the nationality of the cook and whether seafarers would buy nuts or pistachios that are not salted if they were available in the shop on board.

The cook questionnaire additionally asked how many cooking training courses offered by the employer the ship cooks had participated in, when the last one had taken place and whether additional training courses about the country-specific cuisine of other seafarers’ nationalities would be helpful. Furthermore, it was asked about the use of cookbooks and recipe collections for cooking on board and whether the cook takes food preferences of different nationalities into account when preparing food. Lastly, detailed questions about for which tasks the ship cooks would potentially use a digital platform on a newly provided tablet that could support them with their daily and complex tasks and whether they could imagine applying and implementing the principles of nudging on board. A detailed presentation of all questions and answer scales can be found in Table S1.

### Participants

The questionnaires were sent to 1,005 seafarers on 68 merchant ships of two German shipping companies. A total of 970 seafarers responded (96.5%), of which 62 were cooks completing the cooks’ questionnaire. Of the remaining 908 participants, there were 52 excluded due to missing information on nationality (37) or gender (12), or since they did not respond to any of the surveyed questions (3). In addition, in the crew questionnaire, 39 seafarers were excluded from Ethiopia (17), India (15), Sri Lanka (4), Turkey (1), China (1) and Georgia (1) from analyses as their nationalities each had too few participants for a meaningful statistical comparison. There were seven female participants with the same reason for exclusion.

For statistical analysis of the crew questionnaire, the remaining 810 seafarers were grouped according to their nationality. All tabulations compare the group of South-East Asians (SE Asians; 533), which consists of seafarers from the Philippines (505) and Burma (28), with Europeans (277), who originated from Ukraine (74), Romania (67), Poland (60), Russia (26), Germany (13), Bulgaria (11), Lithuania (9), Estonia (4), Portugal (3), Montenegro (3), Slovakia (3), Europe (2), Hungary (1) and Croatia (1). The cook questionnaire was completed by 62 of the 68 participating ship cooks (91.2% response), including Filipinos (58), Burmese (2) and Ukrainians (2).

### Statistical analysis

Categorical variables were presented by their absolute and relative frequencies and continuous variables by mean and standard deviation. Group differences were examined by using Chi-square tests and t-tests as appropriate. Due to the descriptive nature of this work, missing values were not addressed for the largest part of the statistical analysis, however, the N-values for each question are given in tables and missing values are shown in graphs. For correlations and regression analyses, cases were excluded listwise.

Pearson correlations of variables were applied. The size of each significant correlation coefficient was interpreted as per rule of thumb (negligible: *R* < .3, low: 0.3 < *R* < .5, moderate: 0.5 < *R* < .7, high: 0.7 < *R* < .9, very high: 0.9 < *R* < 1.0) [15]. To analyse the association of seafarers’ attitudes towards healthy food and environmental factors influencing food choices with seafarer’s nationality, binary logistic regression analyses were performed. To avoid the effects of multicollinearity, the variable of job experience was excluded from all models as it correlated with age (*R* = .876, *p* < .001). The crude models displayed in Table 1 equal a Pearson correlation (unadjusted). Adjusted models were applied for age, Body Mass Index (BMI), officer, working area on board and vessel type. The BMI was calculated according to the World Health Organization: BMI = (body weight in kg) / (body height in m)^2^ [16]. Underweight was defined as BMI up to 18.5 kg/m², normal weight as BMI ≥ 18.5 kg/m² up to 25 kg/m², overweight as BMI ≥ 25 kg/m² up to 30 kg/m² and obesity as BMI ≥ 30 kg/m². All statistical tests were computed using SPSS Statistics v. 26.0 (IBM, Armonk, NY, USA) and Microsoft Excel 2013 (Microsoft, Redmond, WA, USA) with the significance level set to *a* < 0.05.

**Table 1:** Baseline characteristics for the seafarers responding to the crew questionnaire (N = 810)

	N	Total		European (N = 277)		SE Asian (N = 533)	Test Statistic	p
		N (%) / M (SD)	N	N (%) / M (SD)	N	N (%) / M (SD)		
Age (in years)	798	38.6 (10.3)	275	39.4 (11.7)	523	38.1 (9.4)	1.6	.108^a^
Job experience (in years)	769	14.2 (9.4)	263	16.6 (11.0)	506	12.9 (8.2)	4.9	< .001^a^
Officer	807	321 (39.8%)	277	256 (92.4%)	530	65 (12.3%)	487.9	< .001^b^
Vessel type	799		274		525		4.2	.249^b^
Container		559 (70.0%)		190 (69.3%)		369 (70.3%)		
Bulk carrier		201 (25.2%)		65 (23.7%)		136 (25.9%)		
Other		39 (4.9%)		19 (6.9%)		20 (3.8%)		
Working Area	807		277		530		77.0	< .001^b^
Deck		338 (41.9%)		170 (61.4%)		168 (31.7%)		
Engine		425 (52.7%)		107 (38.6%)		318 (60.0%)		
Galley*		44 (5.5%)		0 (0%)		44 (8.3%)		
BMI (in kg/m^2^)	785	25.6 (3.5)	271	26.3 (3.1)	514	25.2 (3.6)	4.3	< .001^a^
BMI Group	785		271		514		21.8	< .001^b^
Underweight		7 (0.9%)		1 (0.4%)		6 (1.2%)		
Normal weight		345 (43.9%)		90 (33.2%)		255 (49.6%)		
Overweight		357 (45.5%)		150 (55.4%)		207 (40.3%)		
Obesity		76 (9.7%)		30 (11.1%)		46 (8.9%)		

*Note* SE Asian = South East Asian; BMI = Body Mass Index

*Galley personnel only consists of messmen as cooks were excluded from this questionnaire. Values are given as numbers (percentage) or mean (standard deviation). ^a^t-test. ^b^chi-squared test

## Results

### Demographic and occupational characteristics of the crew questionnaire

European seafarers were 39.4 (11.7) years old on average and therefore not significantly older than SE Asian seafarers, who were 38.1 (9.4) years old. However, European seafarers reported significantly higher job experience (t = 4.9; *p* < .001) and were more likely to be officers (z = 487.9; *p* < .001). Furthermore, 41.9% of seafarers stated working on deck (61.4% Europeans vs. 31.7% SE Asians), 52.7% in the engine room (38.6% Europeans vs. 60.0% SE Asians) and 5.5% in the galley (0.0% Europeans vs. 8.3% SE Asians, cooks excluded). The average BMI was significantly higher for European than for SE Asian seafarers (t = 4.3; *p* < .001) and of all seafarers 45.5% were overweight (55.4% Europeans vs. 40.3% SE Asians) and 9.7% were obese (11.1% Europeans vs. 8.9% SE Asians) (Table 2.).

**Table 2:** Association of seafarers’ attitude towards healthy food and environmental factors influencing food choices with seafarer’s nationality

	Total (N = 810)	European (N = 277)	SE Asian (N = 533)	Crude Test Statistic		Adj. Test Statistic^1)^		
	N (%)	N (%)	N (%)	OR (95% CI)	p	OR (95% CI)	p	Nagelkerke R^2^
For my well-being, it is important for me to eat healthy food.	795 (98.8%)	269 (98.2%)	526 (99.1%)	0.31 (0.07, 1.32) ^2)^	0.114	0.09 (0.01, 0.73)	0.025	0.69
I would be willing								
to change my eating habits	685 (88.4%)	215 (83.7%)	470 (90.7%)	0.55 (0.33, 0.84)	0.007	0.31 (0.14, 0.73)	0.007	0.70
to eat less meat	635 (80.0%)	149 (56.0%)	486 (92.0%)	0.11 (0.07, 0.16)	< 0.001	0.11 (0.05, 0.22)	< 0.001	0.74
to eat more vegetables	788 (98.3%)	260 (95.9%)	528 (99.4%)	0.14 (0.04, 0.50)	0.005	0.04 (0.01, 0.27)	0.001	0.71
to eat more fruits	792 (98.6%)	264 (97.1%)	528 (99.4%)	0.19 (0.05, 0.73)	0.015	0.06 (0.01, 0.51)	0.011	0.70
If superiors are good examples for healthy life style on board, it is likely that I will follow their behavior.	722 (92.4%)	208 (81.9%)	514 (97.5%)	0.11 (0.06, 0.22)	< 0.001	0.06 (0.02, 0.17)	< 0.001	0.71
If the cook on board is from the same culture as myself, it is more likely that I am satisfied with the meals.	678 (86.3%)	180 (70.3%)	498 (94.0%)	0.15 (0.10, 0.24)	< 0.001	0.18 (0.09, 0.39)	< 0.001	0.70
I would buy nuts or pistachios that are not salted, if available in the shop.	610 (78.1%)	194 (71.9%)	416 (81.4%)	0.60 (0.42, 0.85)	0.004	0.40 (0.22, 0.73)	0.003	0.69

*Note* Values are given as numbers (percentage). Results of binary logistic regressions are described as Odds Ratios by 95% confidence interval OR (95% CI), p-value and Nagelkerke R^2^

The nationality of SE Asia was 0 and European was coded 1

1) Adjusted for age, body mass index, officer, working area on board and vessel type

2) Omnibus tests of model coefficients are not significant and thus, the model is not improved by adjustment

### Seafarers’ attitudes towards healthy food and environmental factors on board that may influence food choice positively

According to 98.8% of all seafarers, it is important for their well-being to eat healthy food (98.2% Europeans vs. 99.1% SE Asians). Regarding the willingness to adapt eating habits, 88.4% of the seafarers agreed, although significantly more SE Asians than European seafarers (83.7% Europeans vs. 90.7% SE Asians; *p* = .007). This also applies to the willingness to eat more vegetables (95.9% Europeans vs. 99.4% SE Asians; *p* = .005) and fruits (97.1% Europeans vs. 99.4% SE Asians; *p* = .015). In addition, while only 56.0% of European seafarers were willing to eat less meat, significantly more SE Asians agreed to do so (92.0%; *p* < .001). Asked whether they would follow superiors’ behavior, if they were good examples for healthy lifestyle on board, 92.4% of seafarers agreed (81.9% Europeans vs. 97.5% SE Asians; *p* < .001). Furthermore, 86.3% of seafarers reported that they would be more satisfied with the meals on board if they are prepared by a cook from the same culture (70.3% Europeans vs. 94.0% SE Asians; *p* < .001). Additionally, 81.4% of SE Asian and 71.9% of European seafarers responded, they would purchase them from the onboard duty free shop, if salted nuts or pistachios were available. The results of the logistic regressions in Table 1 demonstrate that all questions except for the first were significantly associated with SE Asian seafarers.

### Demographic and occupational characteristics of the cook questionnaire

Of the 62 cooks surveyed, 58 (93.5%) were from the Philippines, 2 (3.2%) from Myanmar and 2 (3.2%) from Ukraine. The average age of the cooks was 44.2 (7.6) years with an average work experience of 17.1 (8.0) years. 45 (72.6%) cooks worked on container vessels, 14 (22.6%) on bulk carriers, 2 (3.2%) on tankers and 1 (1.6%) on another type of vessel. The average BMI was 25.4 (3.0) kg/m^2^ and of all cooks, 48.4% were overweight and 8.1% were obese (Table 3).

**Table 3:** Baseline characteristics for the cook questionnaire (*N* = 62)

	N (%) / M (SD)
Nationality	
Philippines	58 (93.5%)
Myanmar	2 (3.2%)
Ukraine	2 (3.2%)
Age (years)	44.2 (7.6)
Job experience (years)^1^	17.1 (8.0)
Vessel type	
Container	45 (72.6%)
Tanker	2 (3.2%)
Bulk carrier	14 (22.6%)
Other	1 (1.6%)
BMI (kg/m^2^)	25.4 (3.0)
BMI Groups	
Normal weight	27 (43.5%)
Overweight	30 (48.4%)
Obesity	5 (8.1%)

*Note*^1^N=61. Values are given as numbers (percentage) or mean (standard deviation)

### Availability of possible support approaches for the cook

**Fig. 1:**
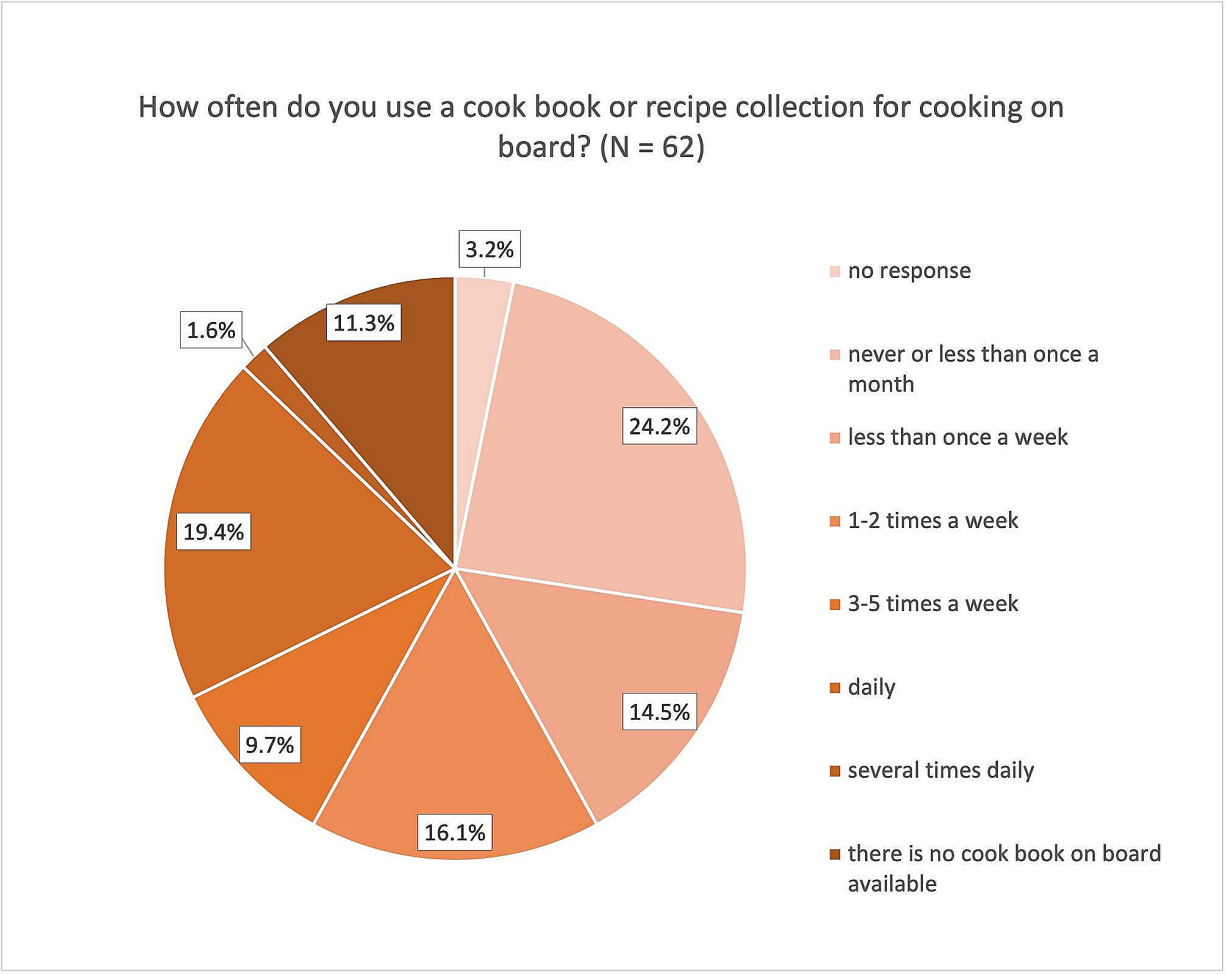
Use of cookbooks or recipe collections

The use of cookbooks or recipe collections varies widely. While 19.4% of the ships’ cooks stated that they use such aids daily, 24.2% answered that they never or less than once a month use a cookbook or recipe collection (Fig. 1). 11.3% even answered that no cookbook or recipe collection was available on board. When asked whether the food preferences of the different seafarer nationalities are taken into account for food preparation, all the ship cooks surveyed, agreed.

**Fig. 2:**
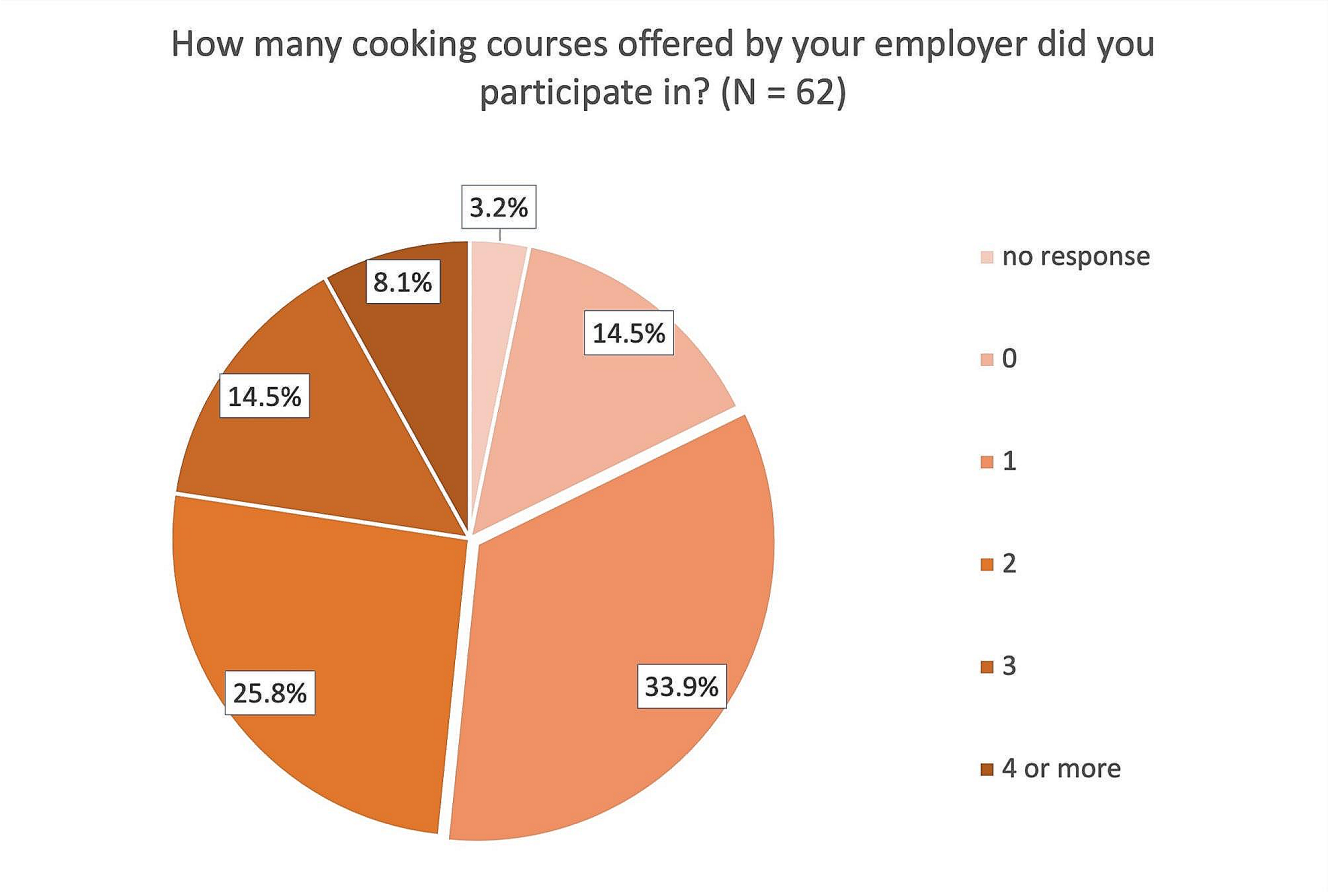
Participation in cooking courses offered by the employer

**Fig. 3:**
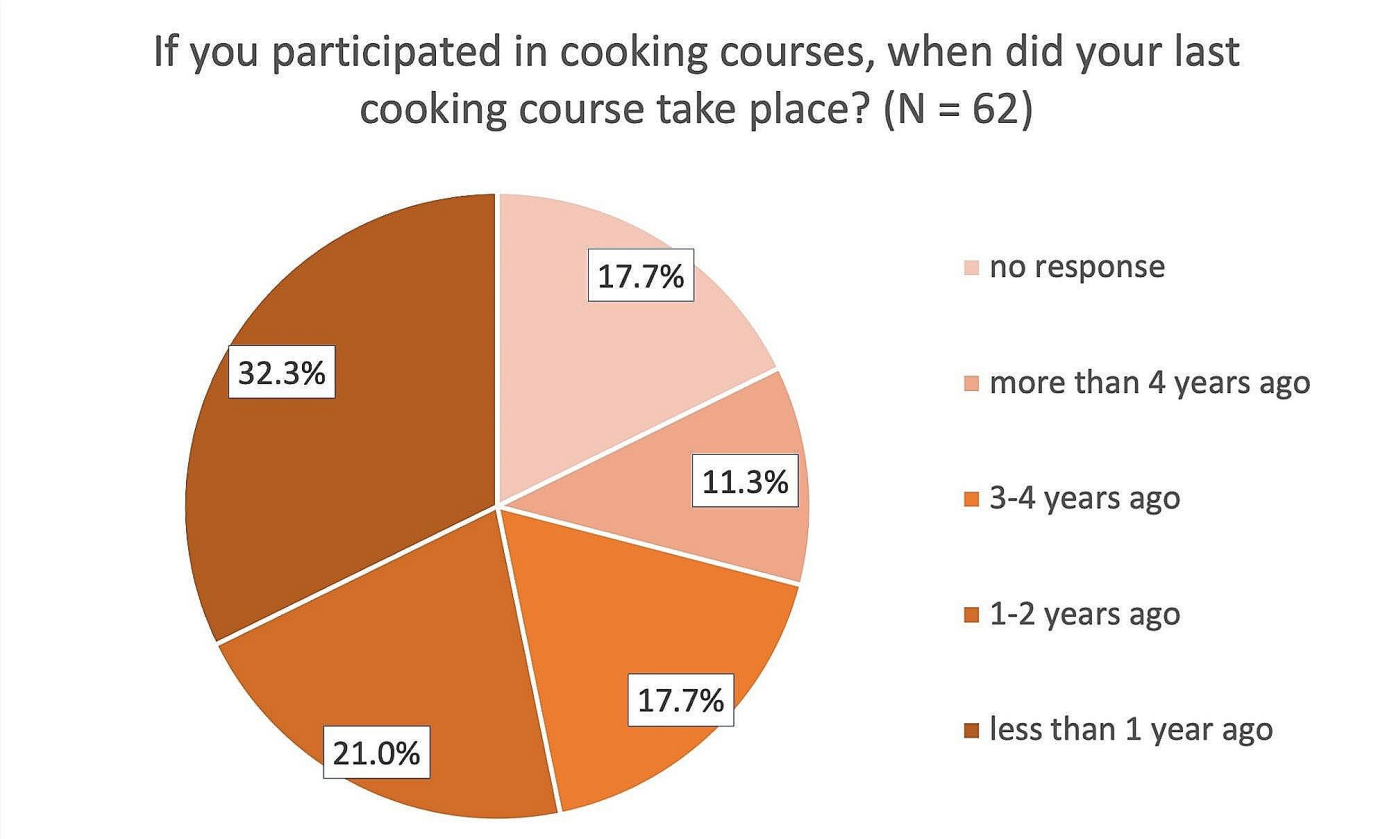
Last participation in a cooking course

While 82.3% of the ship cooks reported having taken part in at least one cooking course organized by their employer (1: 33.9%, 2: 25.8%, 3: 14.5%, 4 or more: 8.1%), 14.5% also reported not having taken any cooking courses (Fig. 2). Slightly above half reported that the last of these courses had taken place no more than 2 years ago (Fig. 3). In addition, 61 of the 62 ship cooks surveyed agreed that an additional cooking course about the country-specific cuisine of other seafarer nationalities would help them with their daily work on board.

### The ship cooks’ attitudes towards interventional approaches

**Fig. 4:**
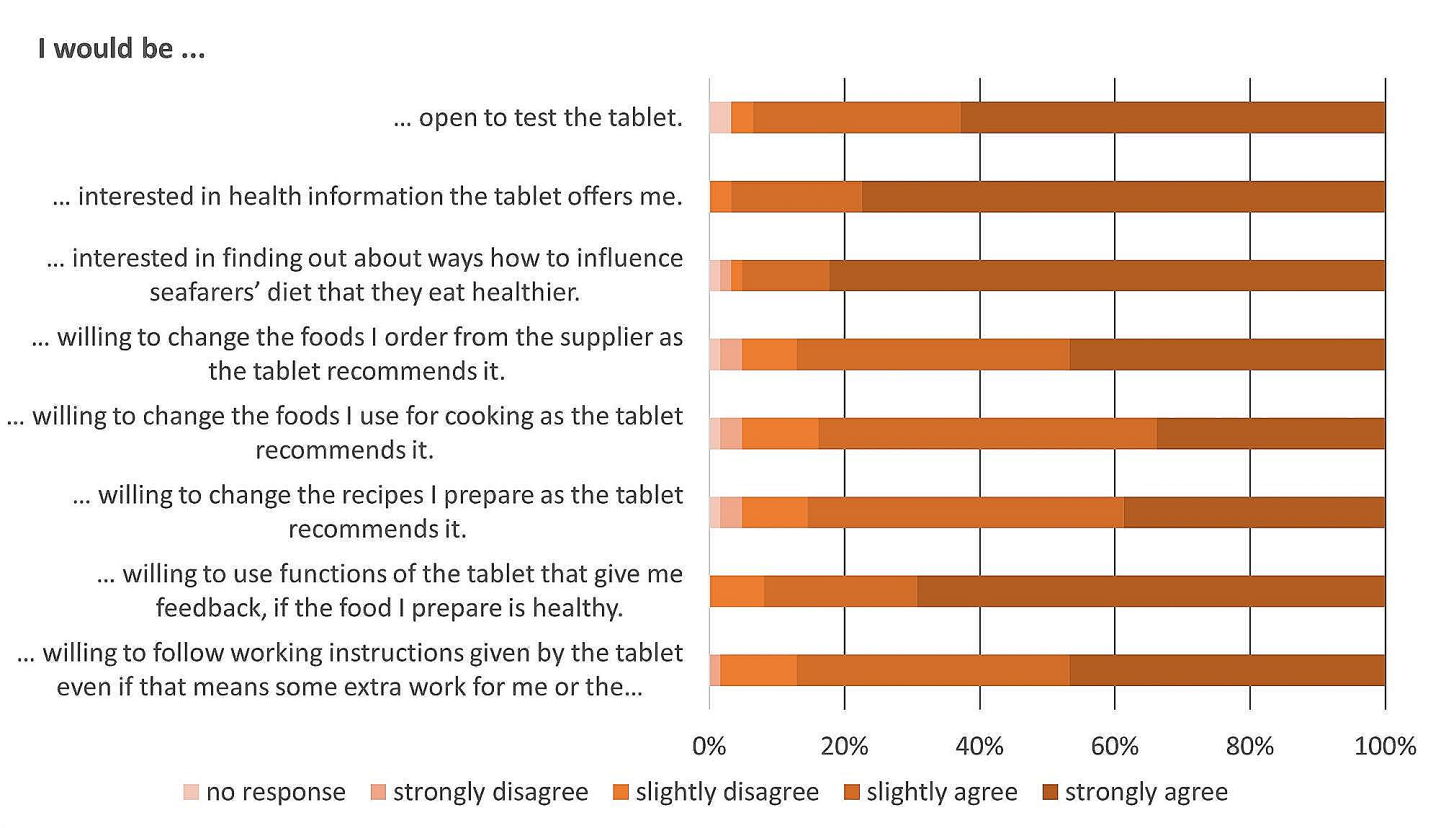
Attitudes towards a tablet as an interventional tool to facilitate the cook’s work

The willingness to use a tablet (“slightly to strongly agree”) as a support tool for the ship cooks’ work was high at 93.5%. Interest in possible functions of such a tablet was highest for information about health aspects of nutrition (96.8%), information about nudging measures (95.2%) and a feedback function that evaluates the health aspect of the food prepared by the ship cooks (92.0%). The willingness to use functions that optimize meal ordering (87.1%) or recommend recipes (85.5%) or food (83.9%) for meal preparation was also high. About 87.1% of the cooks said they would be willing to follow the tablet’s instructions, even if it meant extra work for themselves or the messman (Fig. 4).

**Fig. 5:**
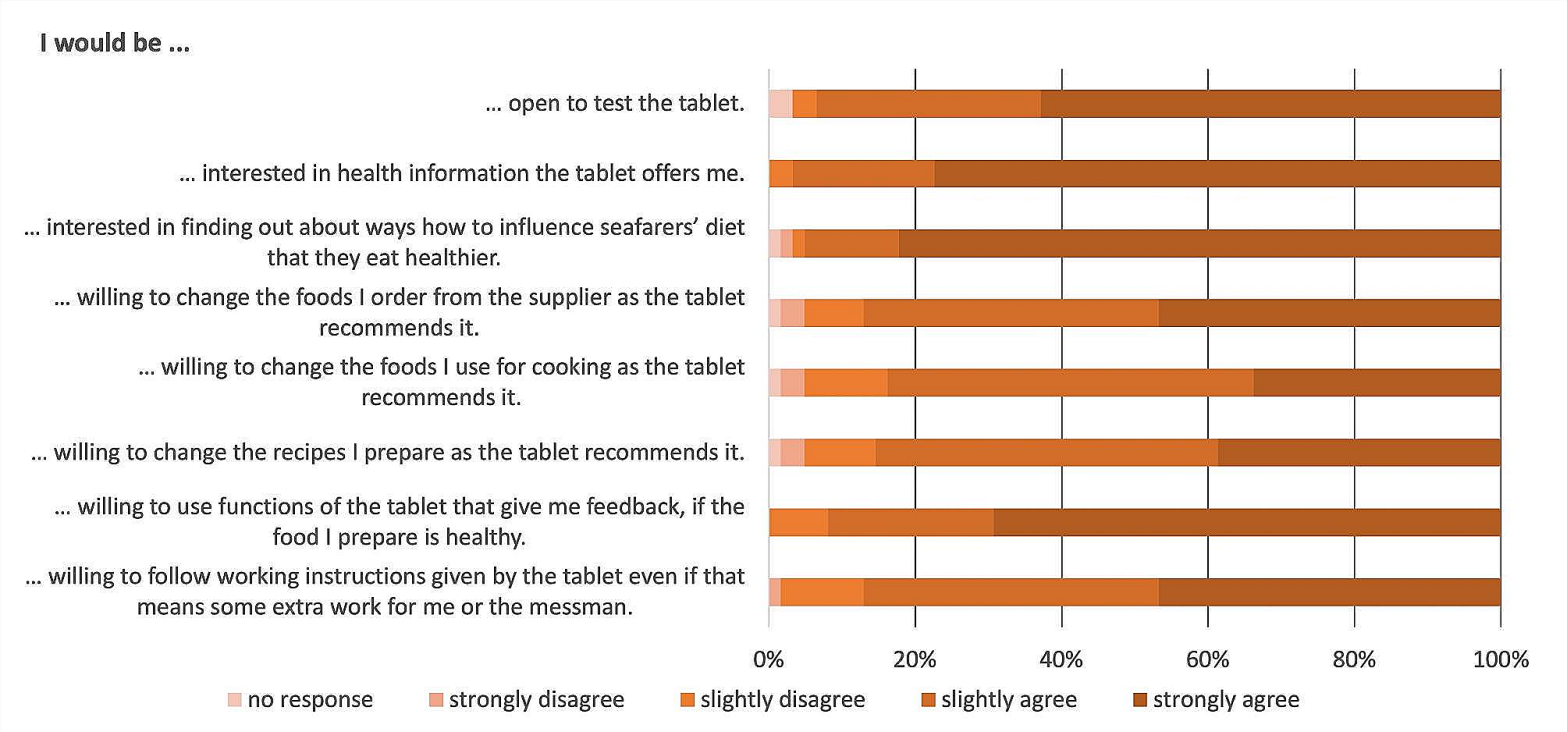
Applicability of an example for nudging

Lastly, 95.1% of the cooks responded that their crew would probably or definitely like the approach of nudging and that either they or the messman have time to prepare the food accordingly (Fig. 5).

## Discussion

To our knowledge, this is the first study to quantitatively investigate seafarers’ and ship cooks’ attitudes and willingness to adopt approaches for improving the nutritional situation on board. According to the crew questionnaire, a large proportion of all seafarers consider healthy eating important for their well-being and are willing to adapt their eating habits. However, the willingness to change is lower among European seafarers than SE Asian seafarers. These differences may be due to the general attitudes of Asian seafarers, who often learned to adapt to and accept new situations on board, while European crew members - most of them officers - often experienced a higher degree of self-determination in their daily work.

The responses of ship cooks on availability and use of cookbooks or recipe collections, as well as participation in cooking classes, varied widely, which could be due to the fact that the cooks seem to be left on their own in the way they work. Overall, ship cooks showed a high willingness and interest in support measures and intervention approaches to improve the nutritional situation on board, both for the use of a tablet and for the principles of nudging.

The current situation concerning seafarers’ nutrition on merchant ships requires improvement. How this can be achieved is still uncertain, especially as there are a variety of barriers [12]. The seafarers in our study reported that healthy food is important for their well-being and that they are willing to adapt their dietary habits. In addition, found that onboard food plays an important role in job satisfaction [17]. Nevertheless, ship cooks reported that a variety of complex factors such as eating traditions, age and hierarchy were barriers to a positive reception of change by the crew [12]. This is in line with the present findings, as in particular older and European seafarers, who mostly hold officer positions on merchant ships, responded to be less willing to accept changes to their nutrition. For example, only 56% of all European seafarers indicated that they would be willing to eat less meat, although the current consumption of fat and meat on board is certainly too high [6, 11]. This is particularly disadvantageous because, on the one hand, our results also show that superiors on board could act as role models and thus could encourage healthier eating habits, and on the other hand because it means that ship cooks can be exposed to social pressure from colleagues and higher-ranked officers. This is supported by a recent qualitative study by Baum-Talmor and Şahin, which reports the statement of a ship cook that he is primarily concerned with keeping the captain, chief officer and chief engineer satisfied, as they have the power to dismiss seafarers from the ship [18].

In a low-stimulus and constant environment like a ship, where changes might be perceived particularly quickly and are not necessarily desired by superiors, it, therefore, seems necessary in terms of feasibility that some adjustments, such as the reduction of meat consumption, must be made in small steps. However, this does not apply to all changes, as both European and SE Asian seafarers were in favour of higher fruit and vegetable consumption. This is not a surprising finding as prior research conducted by the authors has shown that these foods are being less consumed on board than at home due to limited availability [7].

Attempts to counteract the lack of fresh fruits and vegetables, such as consuming food with the shortest shelf-life first and freezing longer-lasting foods, are not sufficient. The limited supply of fruit and vegetables on board is due to various structural factors, such as limited storage space and high purchasing cost [12]. What food gets on board depends on the delivery capacity of the caterers, the location of the port at which the food is loaded and the budget provided by the shipping companies. Shipping companies must provide suitable framework conditions to sustainably change the nutritional situation on merchant ships. Moreover, Babicz-Zielińska and Zabrocki found out that the proportions of different food groups ordered on 55 sea-going vessels and 36 deep-sea fishing ships led to an unfavourable food supply on board [11].

To improve the composition of the stored food, the ordering process, which is currently processed by the ship cook and ship master, must be changed. Especially, as ship cooks are usually not trained to take responsibility for ordering, but are expected to take into consideration the nutritional needs of the seafarers and anticipate logistical difficulties in advance [17]. The same applies to the ship masters, who have to balance the food order demanded by the cook with the often low budgets provided by the shipping companies. An easy approach we suggest that could potentially promote improvement is the modification of food ordering practices, e.g. by introducing modified food ordering forms that set minimum and maximum quantities for certain food groups. This ordering form could also be made available on a digital platform via tablet. This would be an approach welcomed by the ship cooks (87.1% agreement) and above that the editing process could be supported and simplified with the help of a shore-based specialist.

The ship at sea is a largely closed environment in which it would then be difficult to work around the promotion and demand of healthy eating. Hjarnoe and Leppin suggested substituting high-sugar and high-fat foods and eliminating soda drinks at the supply chain level [12]. Also, if feasible, we would establish the supply of mineral-rich water and iodised salt instead of table salt on board. Moreover, improvements could be made in the ship store, which usually provides seafarers with duty-free sweets, snacks, sugar-sweetened beverages and consumables needed for everyday life. Offering healthy foods for purchase independent of the kitchen supply, such as unsalted nuts and pistachios, but also dried fruits and vegetables, and dried fish, could reduce the consumption of unhealthy sweets and snacks.

Finally, each seafarer decides on his or her eating habits, and by far not all seafarers in our survey showed willingness to adjust them. Appropriate education of seafarers about nutrition that enables individuals to make the right choices about healthy eating and portion sizes could be a solution for this. Providing nutrition information via apps or posters and monitors on the messroom walls would contribute to nutrition knowledge and could consequently lead to a higher motivation to switch to a healthy diet. Nevertheless, the food prepared by ship cooks remains the factor that has the greatest impact on seafarers’ nutrition and should therefore receive special attention and support from the shipping companies. Cooks must be well-trained to do their job to the best of their ability. Not only are they responsible for the majority of the food consumed by seafarers, but also challenged to satisfy everyone on board by taking the multinational crews with different preferences into account [17]. To support this, ship cooks should have the possibility to regularly take cooking courses, but instead 14.5% of the cooks we surveyed had never attended a cooking course offered by their employer, and for almost half of the cooks the last course was three years or more in the past.

In addition, 61 out of 62 cooks reported that more courses on the country-specific cuisine of other seafarer nationalities would help them in their daily work. It seems that the ship cooks in our survey were inadequately trained and supported in the carrying out of their duties. A well-trained cook could not only prepare nutritious meals but also implement intervention measures. Raising awareness of quick-to-implement nudging methods, such as offering cut fruit and vegetables instead of biscuits at coffee break, or making invisible changes, such as reducing fat and sugar in familiar foods, could be an effective way to improve nutrition on board [14]. Above all this, our results also show that this is a way that enjoys a high level of acceptance among the ship cooks we surveyed and thus is apt to improve job satisfaction. A gradual adjustment to the recommended portion sizes of the healthy eating plate over several weeks is another approach according to the principle of nudging that could be explored [19].

A recent study found that seafarers have a high level of IT proficiency and thus their technical competence would be adequate for app-based health promotion [20]. As such, digital platforms delivered on a work tablet or smartphone represent an interesting way to deliver content, such as nudging methods or cooking training, directly to the cooks. Pictures and videos that explain the reasoning and application of these measures, as well as a success control after implementation, could increase the ship cooks motivation to make such changes. However, a digital platform has even more potential, as, it could provide recipe collections and cookbooks. These are regularly used by a relevant number of ship cooks but are not available on every ship. Also, as mentioned earlier, the food ordering process could be enhanced. In general, the ship cooks in our survey were open-minded for the use of a digital tool with at least 85% agreement for all areas of application queried, so that a technically well-implemented digital platform seems promising in many respects.

### Limitations

Of course, there are also limitations to our study. Firstly, combining European seafarers from different countries into one group was necessary to allow for evaluation so that cultural differences between different European nationalities may confound the results for this group. Secondly, the results and conclusions are limited to male seafarers of the nationalities studied. Thirdly, the seafarers surveyed worked exclusively on ships of two German shipping companies, which is why the results cannot be generalised. Finally, this study is based on a cross-sectional approach, which excludes the possibility of cause-and-effect interpretations.

## Conclusion

To improve nutrition on board merchant ships and thus support the health of seafarers, it is necessary to adjust various parameters, from ordering procedures to the consumption decisions of the individual. The shipping companies are required to take on more responsibility and to find ways of exerting a positive influence. The ship cooks can play a decisive role in this, as they have the greatest influence on onboard catering, apart from the limiting prevailing framework conditions. Regular training for ship cooks is a certain expected minimum, which, however, is not always met. There are various ways to support ship cooks, but so far only cooking courses have been scientifically evaluated.

The development of a digital platform delivered on a tablet that supports the ship cooks in daily and complex tasks, such as menu planning or food ordering, offers training and empowers them to implement health-promoting measures themselves, seems to be an accepted and, at least in theory, promising measure to implement strategic, health-promoting changes in the ship environment. However, this approach needs to be tested in intervention studies to evaluate its suitability for improving seafarers’ nutrition, which is critical to their overall health and well-being during long periods at sea.

### Electronic supplementary material

Below is the link to the electronic supplementary material.

Supplementary Material 1

## Data Availability

The datasets used and/or analysed during the current study are available from the corresponding author upon reasonable request.

## Abbreviations

BMI: Body Mass Index
SE Asian: Southeast Asian

## References

[CR1] 1. von Katzler R, Zyriax B-C, Jagemann B et al. Lifestyle behaviour and prevalence of cardiovascular risk factors - a pilot study comparing Kiribati and European seafarers. BMC Public Health 2019;19:855. 10.1186/s12889-019-7186-2 PMC6604182 31262273

[CR2] 2. Roberts SE, Jaremin B. Cardiovascular disease mortality in British merchant shipping and among British seafarers ashore in Britain. Int Marit Health 2010;62:107–116. 21154296

[CR3] 3. Oldenburg M. Risk of cardiovascular diseases in seafarers. Int Marit Health 2014; 65:53–57. 10.5603/IMH.2014.0012 25231325

[CR4] 4. Hansen HL, Hjarnoe L, Jepsen JR. Obesity continues to be a major health risk for Danish seafarers and fishermen. Int Marit Health 2011;62:98–103. 21910112

[CR5] 5. Møller Pedersen SF, Riis Jepsen J. The metabolic syndrome among Danish seafarers. Int Marit Health 2013;64:183–190. 10.5603/imh.2013.0002 24408138

[CR6] 6. Zyriax B-C, von Katzler R, Jagemann B et al. Food offerings on board and dietary intake of European and Kiribati seafarers - cross-sectional data from the seafarer nutrition study. J. Occup. Med. Toxicol 2018;13:9. 10.1186/s12995-018-0190-0 PMC6034640 29988947

[CR7] 7. Neumann FA, Belz L, Dengler D et al. Eating behaviour and weight development of European and Asian seafarers during stay on board and at home. J. Occup. Med. Toxicol 2021;16:41. 10.1186/s12995-021-00329-9 PMC8439037 34521438

[CR8] 8. Hjarnoe L, Leppin A. A risky occupation? (Un)healthy lifestyle behaviors among Danish seafarers. Health Promot Int 2014a;29:720–729 10.1093/heapro/dat024 23630132

[CR9] 9. Jezewska M, Babicz-Zielińska E, Leszczyńska I, Grubman M. Promotion of healthy nutrition of seafarers. Int Marit Health 2009;60:48–50. 20205129

[CR10] 10. Baygi F, Mohammadi-Nasrabadi F, Zyriax B-C et al. Global overview of dietary outcomes and dietary intake assessment methods in maritime settings: a systematic review. BMC Public Health 2021;21:1579. 10.1186/s12889-021-11593-z PMC8379789 34419000

[CR11] 11. Babicz-Zielińska E, Zabrocki R. Assessment of nutrition of seamen and fishermen. Rocz. Panstw. Zakl. Hig 1998;49:499–505. 10224895

[CR12] 12. Hjarnoe L, Leppin A. What does it take to get a healthy diet at sea? A maritime study of the challenges of promoting a healthy lifestyle at the workplace at sea. Int Marit Health 2014b;65:79–86. 10.5603/IMH.2014.0018 25231331

[CR13] 13. Wyse R, Jackson JK, Delaney T et al. The effectiveness of interventions delivered using digital food environments to encourage healthy food choices: A systematic review and meta-analysis. Nutrients 2021;13 doi: 10.3390/nu13072255. 10.3390/nu13072255 PMC8308236 34208869

[CR14] 14. Westenhoefer J, von Katzler R, Jensen H-J et al. Cultural differences in food and shape related attitudes and eating behavior are associated with differences of Body Mass Index in the same food environment: cross-sectional results from the Seafarer Nutrition Study of Kiribati and European seafarers on merchant ships. BMC Obesity 2018;5:1. 10.1186/s40608-018-0180-x PMC5784660 29416869

[CR15] 15. Hinkle DE, Wiersma W, Jurs SG. Applied Statistics for the Behavioral Sciences. 5th Edition. Boston: Houghton Mifflin Company, 2003.

[CR16] 16. World Health Organization (WHO). A healthy lifestyle - WHO recommendations. 2010. Available at: https://www.who.int/europe/news-room/fact-sheets/item/a-healthy-lifestyle---who-recommendations (last accessed 24 July 2023).

[CR17] 17. Oldenburg M, Baur X, Schlaich C. Occupational risks and challenges of seafaring. J. Occup. Med. Toxicol 2010;52:249–256. 10.1539/joh.k10004 20661002

[CR18] 18. Baum-Talmor P, Şahin ÇE. Employment Practices, Cost Minimization, and Their Implications for Food Provisions and Seafarers’ Wellbeing on board Ships - A Qualitative Analysis. Inquiry. 2024;61:469580241229613. 10.1177/00469580241229613. 10.1177/00469580241229613 PMC10832409 38297888

[CR19] 19. Harvard School of Public Health. Healthy Eating Plate. Harvard School of Public Health. 2023. Available at: https://www.hsph.harvard.edu/nutritionsource/healthy-eating-plate (last accessed 17 July 2023).

[CR20] 20. Arslan LC, Dengler D, Belz L et al. Exploration of Seafarers’ Mobile Proficiency as a Prerequisite for Possible Health App-based Health Promotion on Board. Inquiry. 2023;60:469580231206264. 10.1177/00469580231206264. 10.1177/00469580231206264 PMC10621288 37909669

