# Supplementary material for: Seafarers’ attitudes and chances to improve the nutrition on merchant ships from the crews’ and cooks’ perspective
Source: J Occup Med Toxicol. 2024 May 2;19:13. doi: 10.1186/s12995-024-00412-x (PMC11067207; doi:10.1186/s12995-024-00412-x)
Supplement: Supplementary file 1 — Supplementary Material 1 [file 12995_2024_412_MOESM1_ESM.docx]

**Seafarers’ attitudes and chances to improve the nutrition on merchant ships from the crews’ and cooks’ perspective**

By Felix Alexander Neumann, Lukas Belz, Dorothee Dengler, Volker Harth, Chiara Reck, Marcus Oldenburg, Birgit-Christiane Zyriax

# Supplement

**Table S1** Questionnaire

| **Demographic Data** | |
| --- | --- |
| **Questions** | **Response Options** |
| 1. What is your age? | [years] |
| 2. What is your rank? | Nautical officer, Technical officer, Rating engine, Rating deck, Galley staff, Cadet |
| 3. What is your nationality? |  |
| 4. What is your gender? | Male, Female, Other |
| 5. What is your height? | [meter or foot and inches] |
| 6. What is your actual weight? | [kilogram or pound] |
| 7. For how many years have you been a seafarer? | [years] |
| 8. What kind of vessel are you working on right now? | Container vessel, Tanker, Bulk carrier, Supplier, Passenger vessel, Fishery vessel, Other |
| **Crew Questionnaire** | |
| **Questions** | **Response Options** |
| 9. For my well-being, it is important for me to eat healthy food. | *Agree, Disagree* |
| 10. I would be willing to change my eating habits. | *Agree, Disagree* |
| 11. I would be willing to eat less meat. | *Agree, Disagree* |
| 12. I would be willing to eat more vegetables. | *Agree, Disagree* |
| 13. I would be willing to eat more fruits. | *Agree, Disagree* |
| 14. If superiors are good examples for healthy life style on board, it is likely that I will follow their behavior. | *Agree, Disagree* |
| 15. If the cook on board is from the same culture as myself, it is more likely that I am satisfied with the meals. | *Agree, Disagree* |
| 16. I would buy nuts or pistachios that are not salted, if available in the shop | *Yes, No* |
| **Cook Questionnaire** | |
| **Questions** | **Response Options** |
| 17. How often do you use a cook book or recipe collection for cooking on board? | Never or less than once a month, less than once a week, 1-2 times a week, 3-5 times a week, daily, several times daily, there is no cook book or recipe collection on board |
| 18. How many cooking courses offered by your employer did you participate in? | 0, 1, 2, 3, ≥4 |
| 18 a. If you participated in cooking courses, when did your last cooking course take place? | less than 1 year ago, 1-2 years ago, 3-4 years ago, more than 4 years ago |
| 19. An additional cooking course about the country-specific cuisine of other seafarer nationalities would help me for my daily work onboard. | 4-point Likert scale from *disagree* to *agree* |
| 20. For meal preparation: Do you take food preferences of the different nationalities into consideration? | *Yes, No* |
| 21. I would be open to test the tablet. | 4-point Likert scale from *disagree* to *agree* |
| 22. I would be interested in health information the tablet offers me. | 4-point Likert scale from *disagree* to *agree* |
| 23. I would be interested in finding out about ways how to influence seafarers’ diet that they eat healthier. | 4-point Likert scale from *disagree* to *agree* |
| 24. I would be willing to change the foods I order from the supplier as the tablet recommends it. | 4-point Likert scale from *disagree* to *agree* |
| 25. I would be willing to change the foods I use for cooking as the tablet recommends it. | 4-point Likert scale from *disagree* to *agree* |
| 26. I would be willing to change the recipes I prepare as the tablet recommends it. | 4-point Likert scale from *disagree* to *agree* |
| 27. I would be willing to use functions of the tablet that give me feedback, if the food I prepare is healthy. | 4-point Likert scale from *disagree* to *agree* |
| 28. I would be willing to follow working instructions given by the tablet even if that means some extra work for me or the messman. | 4-point Likert scale from *disagree* to *agree* |
| 29. An easy way to positively influence a person’s healthy eating behavior would be placing plates with pre-cut fruits & vegetables on the tables of both mess rooms. Would your crew like this method? | 4-point Likert scale: *definitely not, probably not, probably, definitely* |
| 29a. Would you or the messman have time to prepare this method on normal working days? | 4-point Likert scale: *definitely not, probably not, probably, definitely* |
